# Transcriptomic Analysis Exploring the Molecular Mechanisms of Hanchuan Zupa Granules in Alleviating Asthma in Rat

**DOI:** 10.1155/2021/5584099

**Published:** 2021-07-02

**Authors:** Hailong Yin, Yanbo Fan, Dandan Mu, Fei Song, Fang Tian, Qiang Yin

**Affiliations:** ^1^Research and Development Department, Xinjiang Uygur Pharmaceutical Co., Ltd., Wulumuqi, Xinjiang 830001, China; ^2^Science and Education Department, Wuhan Hospital of Traditional Chinese Medicine, Wuhan, Hubei 430014, China; ^3^Department of Management, Xinjiang Uygur Pharmaceutical Co., Ltd., Wulumuqi, Xinjiang 830001, China

## Abstract

**Objective:**

To investigate the molecular mechanisms of HCZP treatment of asthma.

**Materials and Methods:**

Thirty Sprague Dawley (SD) rats were divided into normal, asthma, and HCZP groups (*n* = 10). The asthma model was sensitized by 1 mg ovalbumin (OVA)/aluminum hydroxide Al(OH)_3_mixture and then challenged with 1% aerosolized OVA for four weeks. Rats in the HCZP group received 10.08 g/kg/d HCZP for four weeks during OVA challenge. Then, lung tissues of rats in each group were collected for RNA sequencing. Moreover, the expression level of some core genes was detected by using western blotting and immunohistochemistry.

**Results:**

Inflammatory cell infiltration and pathological damage of the lungs improved in the HCZP group. Compared with the asthma group (0.049 ± 0.002 mm^2^/mm; 0.036 ± 0.006 mm^2^/mm; and 0.014 ± 0.001 mm^2^/mm), total wall thickness (0.042 ± 0.001 mm^2^/mm), inner wall thickness (0.013 ± 0.001 mm^2^/mm), and smooth muscle layer thickness (0.012 ± 0.001 mm^2^/mm) significantly decreased in the HCZP group. Bioinformatics analysis showed that hub genes such as bradykinin receptor B2 (Bdkrb2) and CD4 molecule (Cd4) had different expression patterns between model and HCZP groups. Two transcription factors, forkhead box Q1 (Foxq1) and nuclear factor of activated T cells 2 (Nfatc2), served important regulatory roles in asthma. Compared with the model group, Bdkrb2 protein expression increased and Nfatc2 protein expression decreased in the HCZP group. *Discussion and Conclusion*. HCZP could alleviate asthma via regulating the expression of several hub genes, which might serve as therapeutic targets for asthma. However, the mechanism of these genes will be studied in the future.

## 1. Introduction

Asthma, a common chronic respiratory disease in children and adults, is characterized by reversible airway inflammation obstruction and bronchial hyperresponsiveness [1]. Approximately 315 million people worldwide are affected by asthma, among which about 10% have severe or uncontrolled asthma attacks [2]. Despite the advances in diagnosis and treatment, it remains a serious global health problem [3]. The long-term goal of asthma management is to achieve asthma control and minimize the risk of exacerbation [4]. Currently, pharmacotherapy of asthma mainly includes bronchodilators and anti-inflammatory drugs, such as *β*_2_-agonists and glucocorticoids [5]. However, previous evidence indicates that *β*_2_-agonists are associated with an increased risk of myocardial infarction, congestive heart failure, and sudden cardiac death [6]; meanwhile, glucocorticoids possess extensive immunosuppressive activity and potentially serious side effects and may further promote human metapneumovirus infection which is capable of eliciting inflammatory responses [7]. Thus, it is necessary to explore safe and effective drugs for asthma treatment.

Traditional Chinese medicine (TCM), widely reported to be a complementary and alternative therapy for asthma attacks, can alleviate airway hypersensitivity and inflammatory cell infiltration in patients with asthma [8, 9]. Hanchuan Zupa granule (HCZP) is a Uyghur medical compound preparation produced by modern technology on the basis of Hanchuan Zupa powder in the classic Uyghur medicine “Ruyi Chufang” [10]. It is composed of hyssop (*Hyssopus officinalis* L. (Labiatae), 75 g), adiantum (*Adiantum capillus-veneris* L. (Adiantaceae), 75 g), licorice (*Glycyrrhiza uralensis* Fisch. (Leguminosae), 70 g), fennel (*Foeniculum vulgare* Mill. (Umbelliferae), 125 g), celery seeds (*Apium graveolens* L. (Umbelliferae), 125 g), fenugreek (*Trigonella foenum-graecum* L. (Leguminosae), 75 g), remote lemongrass (*Cymbopogon distans* (Nees) Wats. (Gramineae), 75 g), rugosa rose (*Rosa rugosa* Thunb. (Rosaceae), 75 g), and nettle seed (*Urtica fissa* E. Pritz. (Urticaceae), 70 g) [10]. As a bronchomucotropic agent, HCZP has been widely used in the treatment of the common cold, wind-cold cough, and mucus hypersecretion in asthma [11]. In clinical experiment, HCZP was shown to play a significant role in improving lung function and reducing airway inflammation in asthma patients [12, 13]. However, the systematic study of the effect of HCZP on asthma and its potential molecular mechanism are still limited.

Transcriptome sequencing techniques are widely used in molecular biology research [14]. Yick et al. [15] explore the cellular and molecular pathways in asthma using transcriptomic analysis (RNA-seq). They found that asthma group and normal group had different transcriptomic profiles; in addition, genes such as pendrin and periostin were differentially expressed between asthma and normal, which might be relevant for the pathogenesis and treatment of disease. Nevertheless, genes involved in the HCZP treatment in asthma have not been discovered. Nowadays, the application of RNA-seq in the study of TCM attracts more and more attention of researchers. Due to the lack of genomic data and gene sequence information, the medicinal plants need a large amount of genetic information to analyze the gene function in the whole level [16]. At present, the whole-genome sequencing of most herbs cannot be detected, so it is a quick way to compare the gene sequences and identify the expressed genes by constructing the transcription database [17]. For instance, Jiao et al. [18] revealed the anti-inflammation mechanism of Ma Huang Tang on acute bronchial asthma in mice using RNA-seq. Therefore, in the present study, we constructed a rat model of asthma to investigate the mechanism of HCZP treatment in asthma. The lung tissues from rat in the normal, model, and HCZP groups were respectively extracted and used for RNA-Seq. Our study may provide new insights into the molecular mechanisms of HCZP treatment and provide candidate biomarkers for targeted therapy of asthma.

## 2. Materials and Methods

### 2.1. Animals

Thirty specific-pathogen-free (SPF) male Sprague Dawley (SD) rats weighing 160–200 g were purchased from the Laboratory Animal Center of Hubei Provincial Center for Disease Control and Prevention (Wuhan, China). The rats were kept in the Laboratory Animal Center of Wuhan Hospital of Traditional Chinese Medicine (Wuhan, China) under standard condition (12 h light/dark cycle and 21 ± 1°C) and were acclimated to the conditions for 7 days before the experiment. All animal protocols were approved by the Ethics Committee and Animal Management Committee of Wuhan Hospital of Traditional Chinese Medicine (Approved on 2017-06-02), which conformed to the Guide for the Care and Use of Laboratory Animals published by the US National Institutes of Health [19].

### 2.2. Model Establishment and Drug Treatment

The rats were randomly divided into three groups: normal, model, and HCZP groups, with 10 rats in each group. HCZP was provided by the Preparation Center of the Base of Traditional Chinese Medicine of Wuhan Hospital of Traditional Chinese Medicine. In this study, the dose of HCZP administered to each rat was selected based on the clinical use of HCZP. Specifically, human doses of HCZP were converted to rats doses according to the human-rat coefficient (0.018) of skin surface area [20]; thus, HCZP was administered intragastrically at a dose of 10.08 g/kg. Rats in model and HCZP groups were sensitized on day 1 and day 8 by intraperitoneal injection with 1 mL of OVA/Al(OH)3 normal saline mixture (containing 10 mg OVA and 100 mg Al(OH)_3_) as described previously [21], while rats in normal group were intraperitoneally injected with 1 mL of normal saline. Subsequently, the rats in model and HCZP groups were challenged with 1% OVA for 30 min every other day from the 15th day through the 42th day; the rats in normal group were atomized with normal saline from day 15 to day 42. Notably, during the stimulus period (from day 15 to 42), rats in the HCZP were intragastrically given HCZP (10.08 g/kg) 1 h before atomization, while rats in the model and normal groups were given equal volume of normal saline.

Following a 4-week treatment, rats in each group were given the last challenge after 24 h of withdrawal. Within 2 h after the final challenge, rats were anesthetized with an intraperitoneal injection of 3% pentobarbital sodium. Then, the lung tissues were extracted and the residual blood on the surface of the tissues was eliminated using normal saline. The left lung was fixed in neutral formalin and stored at 4°C for hematoxylin-eosin (HE) staining and immunohistochemistry (IHC), while the right lung tissues were stored at −80°C for transcriptome sequencing.

### 2.3. H&E Staining

After modeling, the pathological changes in the lungs were observed using H&E staining. Lung tissues were fixed overnight in 4% neutral formalin, embedded in paraffin, and cut into 5 *μ*m sections [22]. The sections were dewaxed with xylene and washed with gradient alcohol and distilled water. Then, they were stained with hematoxylin and eosin. Finally, sections were sealed with neutral gum and photographed under a microscope (Olympus IX73, Tokyo, Japan). Moreover, H&E images were analyzed by Image-Pro plus software. Briefly, the indicators of bronchial basal perimeter (Pbm), total wall area (WAt), inner wall area (WAi), and smooth muscle area (WAm) were measured. These measured values were standardized by Pbm, and WAt/Pbm, WAi/Pbm, and WAm/Pbm represented the total wall thickness, inner wall thickness, and smooth muscle layer thickness, respectively [23].

### 2.4. RNA Sequencing

Total RNA from each lung tissue sample was extracted using TRIzol reagent (code no. 9109; TaKaRa, Japan). The 1% agarose gel was used to detect RNA degradation and contamination. RNA purity was monitored using the NanoDrop 2000 (Thermo Scientific). Then, RNA concentration and integrity were evaluated using Invitrogen Qubit RNA IQ kit (Q33221, Thermo Fisher Scientific in Basingstoke, UK) and Agilent 2100 (Agilent Technologies, Palo Alto, CA, USA), respectively. Then, rRNA from the total RNA was depleted using Ribo-Zero Gold rRNA Removal Kit (Illumina, San Diego, CA, USA) according to the manufacturers' instructions. The treated RNAs were interrupted randomly to 200–300 bp by adding ion solution, and then the fragmented RNAs were used as templates to construct the cDNA library. The average insert size for the paired-end libraries was 300–400 bp. After construction of libraries, the concentration and size of library were determined by fluorescence quantification and Agilent 2100 bioanalyzer (Agilent Technologies), respectively. Finally, the libraries were sequenced on the Illumina HiSeq platform [24].

### 2.5. Data Preprocessing

The sequenced data could contain some low-quality bases and incorrectly sequenced bases. In order to filter out unreliable reads, we used the following steps to control the quality of the raw data: (1) the reads with sequencing joints were removed; (2) when the *N* content of sequencing read exceeded 10% of the base number of read, their paired reads were removed; and (3) when the low-quality (*Q* ≤ 5) base number contained in any sequencing read exceeded 50% of the read base number, paired read was excluded [25].

### 2.6. Screening of Differentially Expressed Genes (DEGs)

Raw data were standardized using the TMM algorithm in the edgeR package (version 3.4, http://www.bioconductor.org/packages/release/bioc/html/edgeR.html) [26] and converted to logCPM value. The differential expression analyses between model vs. normal and HCZP vs. model were performed. Then, the corresponding *P* values and logFC values of genes were obtained. The *P* values were adjusted by the Benjamin–Hochberg method (adj. *P* value) [27]. In addition, genes with adj. *P* value < 0.05 and |logFC| > 1 were defined as DEGs. Furthermore, DEGs in model vs. normal groups as well as model vs. HCZP were integrated using Venn analysis, and the overlapping genes were thought to be related to HCZP treatment.

### 2.7. Functional Enrichment Analysis of DEGs

In order to explore the biological function of genes, the functional enrichment analysis was performed. The Gene Ontology (GO) categories and Kyoto Encyclopedia of Genes and Genomes (KEGG) pathways of HCZP-related DEGs were analyzed by using DAVID (version 6.8, https://david-d.ncifcrf.gov/) [28]. Among these, GO annotation included three categories: “biological process (BP),” “molecular function (MF),” and “cellular component (CC)” [29]. *P*value < 0.05 and count > 3 were defined as the enrichment threshold.

### 2.8. Construction of Protein-Protein Interaction (PPI) Network

The interactions among genes were predicted using the STRING database (version 10.0, http://www.string-db.org/) [30]. The input gene set was genes related to HCZP treatment and the species selection was *Rattus norvegicus* (rats). The DEGs were mapped into STRING to obtain PPIs, and a combined score of >0.7 was set as cut-off threshold. Then, PPI network was visualized by Cytoscape software (version 3.4.0, http://chianti.ucsd.edu/cytoscape-3.4.0/) [31]. Finally, the network topology was analyzed using CytoNCA plugin (version 2.1.6, http://apps.cytoscape.org/apps/cytonca) [32]. Furthermore, the key genes were identified based on three centrality measures, including degree centrality (DC), betweenness centrality (BC), and closeness centrality (CC) [33].

### 2.9. TF Prediction and TF-Target Network Construction

All the TFs with motifs in the rat were searched via JASPAR 2018 database (http://jaspar.genereg.net/) [34]. Then, these TFs were intersected with the HCZP-related DEGs to obtain the differentially changed TFs and corresponding motifs. Furthermore, the binding site analysis of the TFs' motif information and genes' promoter sequence in PPI network were performed using online tool Find Individual Motif Occurrences (FIMO, version 5.0.5, http://meme-suite.org/tools/fimo) [35]. TF targets with *P* value < 0.0001 were selected and then visualized by Cytoscape software.

### 2.10. miRNA-Target Interactions Prediction and miRNA-Target Network Construction

miRNA targets were predicted using four currently available methods from miRWalk 2.0 database (http://zmf.umm.uni-heidelberg.de/apps/zmf/mirwalk2/) [36], including miRWalk, miRanda, miRDB, and Targetscan. The miRNA-target pairs that existed in four databases were selected. Furthermore, miRNAs that simultaneously regulated at least 10 genes were screened, and the miRNA-mRNA network was constructed using Cytoscape software.

### 2.11. Western Blot (WB) Analysis

WB analysis was performed to verify the results of bioinformatics analysis. The lung tissues of rats were homogenized with radioimmunoprecipitation assay (RIPA) buffer (code no. P0013B, Beyotime, Shanghai, China), which involved the addition of phenylmethanesulfonyl fluoride (PMSF, no. ST506, Beyotime) and centrifuging at 10000 g for 10 min. The supernatant was collected, and the concentrations of protein were determined using the bicinchoninic acid (BCA) assay [37]. The extracted proteins were separated on the SDS-PAGE gel and transferred to polyvinylidene fluoride membrane. The membranes were blocked in 5% skim milk (0.75 g milk powder + 15 mL PBS) at 37°C for 2 h and then incubated with the Bdkrb2 (cat. no. sc136216, Santa Cruz, CA, USA), Nfatc2 (cat. no. sc7296), and anti-*β*-actin (cat. no. ab8226, Abcam, Shanghai, China) antibodies at 4°C overnight, respectively. Then, the membranes were incubated with secondary antibody at 37°C for 2 h followed by washing with PBST for six times; immunodetection was performed using ECL system. The images were obtained via TanoImage.

### 2.12. Immunohistochemistry

The 5 *μ*m paraffin slices were used to perform IHC staining. After the sections were dewaxed, the activity of endogenous catalase was inhibited with 3% H_2_O_2_ for 10 min at room temperature. The slices were incubated with PBS containing 10% normal goat serum at 37°C for 30 min to reduce nonspecific adsorption, followed by incubation with anti-Bdkrb2 and anti-Nfatc2 antibodies at 4°C overnight. Then, sections were washed with PBS and incubated with second antibody at 37°C for 30 min [38]. Thereafter, the sections were incubated with SABC at 37°C for 0.5 h and placed in DAB solution for staining [39]. Finally, the slices were sealed with neutral gum and observed and photographed under a microscope.

### 2.13. Statistical Analyses

All data are presented as means ± standard deviation (SD). Statistical analyses and plotting were performed using GraphPad Prism 5 (GraphPad Software, San Diego, CA, USA). Statistical significance of the differences between two groups was evaluated by using a two-tailed Student's *t*-test. The results were considered to be statistically significant at *P* < 0.05.

## 3. Results

### 3.1. H&E Staining Analysis

**Figure 1:**
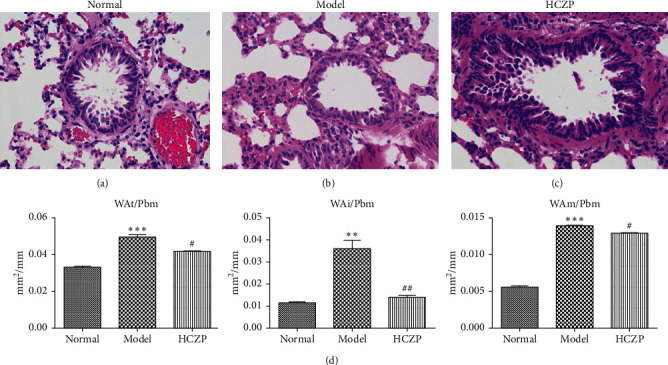
Histopathological changes in rat lung. H&E staining of lung tissues in (a) control group, (b) model group, and (c) HCZP group at magnification × 400. (d) The values of total wall thickness (WAt/Pbm), inner wall thickness (WAi/Pbm), and smooth muscle layer thickness (WAm/Pbm) in three groups. Data are expressed as the mean ± SD (*n* = 10). Data are analyzed using Student's *t*-test. ^*∗∗*^*P* < 0.01 and ^*∗∗∗*^*P* < 0.001 compared with the normal group. #*P* < 0.05 and ##*P* < 0.01 compared with the model group.

After treatment, the histopathological alterations of lung tissues of rats in different groups were observed by H&E staining. Compared with the normal group (Figure 1(a)), H&E staining of the lung tissues from rats in the model group indicated that there was inflammatory cell infiltration around the bronchial wall and blood vessels, which was predominantly composed of eosinophils and lymphocytes (Figure 1(b)). In addition, the epithelial cells were obviously hypertrophic, the bronchial tubes narrowed, and the smooth muscles destroyed in the model group. In contrast, HCZP-treated rats showed alleviated symptoms, indicating pulmonary inflammation, goblet cell proliferation, and mucus secretion were significantly reduced (Figure 1(c)). Moreover, WAt/Pbm, WAi/Pbm, and WAm/Pbm in the model group were significantly higher than those in the normal group (normal vs. model: 0.033 ± 0.001 mm^2^/mm vs. 0.049 ± 0.002 mm^2^/mm; 0.011 ± 0.001 mm^2^/mm vs. 0.036 ± 0.006 mm^2^/mm; and 0.005 ± 0.0003 mm^2^/mm vs. 0.014 ± 0.001 mm^2^/mm). By comparison, WAt/Pbm, WAi/Pbm, and WAm/Pbm in the HCZP group were significantly lower than those of the model group (model vs. HCZP: 0.049 ± 0.002 mm^2^/mm vs. 0.042 ± 0.001 mm^2^/mm; 0.036 ± 0.006 mm^2^/mm vs. 0.013 ± 0.001 mm^2^/mm; and 0.014 ± 0.001 mm^2^/mm vs. 0.012 ± 0.001 mm^2^/mm) (Figure 1(d)).

### 3.2. High-Throughput Sequencing Data

Using high-throughput sequencing, more than 20,000,000 clean reads were generated from each sample. The total clean reads ranged from 6.16 to 7.23 G, the Q30 (%) of these samples was about 90%, and the GC content of samples was less than 50%, suggesting that the quality of the sequencing data was high and could be used for subsequent analysis. According to the sequencing data, a total of 16,818 genes were identified from nine samples, accounting for 78.7–89.9% of mapped reads of all rats genes.

### 3.3. Screening of DEGs

**Figure 2:**
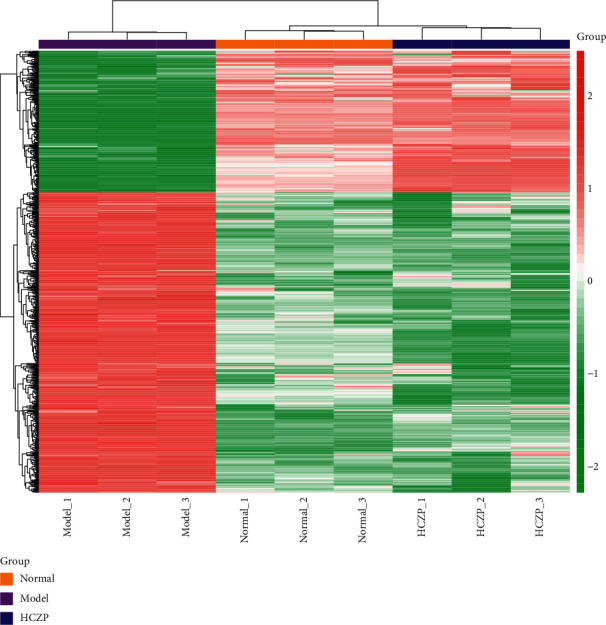
The heatmap of differentially expressed genes in normal, model, and HCZP groups. The *x*-axis represents the samples in different groups. Orange indicates normal group, purple indicates model group, and blue indicates HCZP group. Red to green represent the gene from high expression to low expression.

According to the cut-off criterion of *P* < 0.05 and |logFC| > 1, there were 1,678 DEGs in normal vs. model, including 1,133 upregulated genes and 545 downregulated genes; in addition, 4,461 DEGs were identified between model and HCZP groups, including 1,912 upregulated genes and 2,549 downregulated genes. Moreover, the DEGs in normal vs. model and model vs. HCZP were intersected to identify the genes that were related to HCZP treatment. Finally, a total of 874 DEGs associated with HCZP treatment were obtained (up- or down-regulation of genes was consistent with that in the model vs. normal group). The expression pattern of HCZP-related genes was basically consistent with that of the normal group, which was different in the model group (Figure 2).

### 3.4. GO and KEGG Pathway Analyses of DEGs

**Figure 3:**
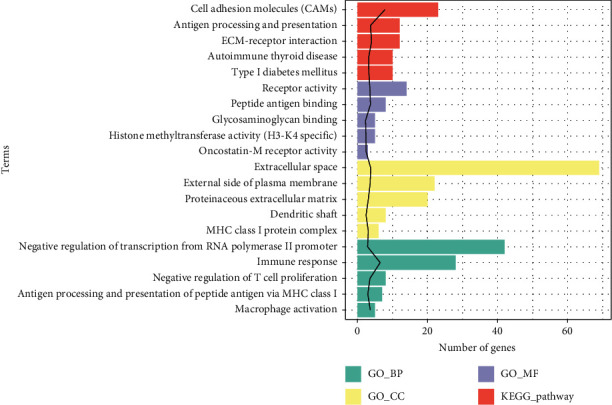
Functional enrichment analysis for DEGs. The black line represents the value of −log10 (*P* value), and the length of the bar represents the number of enriched genes.

GO analysis indicated that DEGs were significantly enriched in 53 GO_BP, 16 GO_MF, and 16 GO_CC. The top five GO terms are shown in Figure 3. In the BP group, DEGs were mainly enriched in immune response (GO: 0006955) and macrophage activation (GO: 0042116); in the MF group, these genes were primarily enriched in antigen-binding peptide (GO: 0042605) and receptor activity (GO: 0004872); in the CC group, the majority of genes were enriched in extracellular space (GO: 0005615) and external side of plasma membrane (GO: 0009897). Furthermore, KEGG analysis revealed that DEGs were significantly involved in 20 pathways. The top five pathways included cell adhesion molecules (CAMs) (rno04514), ECM-receptor interaction (rno04512), antigen processing and presentation (rno04612), type I diabetes mellitus (rno04940), and autoimmune thyroid disease (rno05320) (Figure 3 and Table 1).

### 3.5. PPI Analysis of DEGs

**Figure 4:**
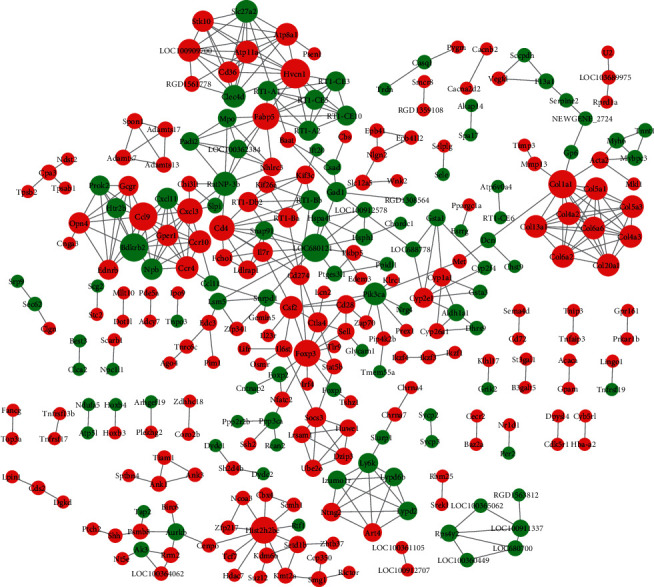
Protein-protein interaction network of HCZP-related DEGs. Red node represents upregulated genes, and green node represents downregulated genes. The larger the node, the higher the degree of connectivity.

The identified DEGs were introduced into the STRING database to obtain the PPI pairs, and these PPIs were then visualized using Cytoscape software. PPI network was composed of 282 nodes and 433 edges (Figure 4). The top 10 nodes with degree scores >10 are listed in Table 2. Among these, Bdkrb2 and CD4 might be regarded as hub genes.

### 3.6. TF-Target Regulatory Network and miRNA-Target Network Construction

**Figure 5:**
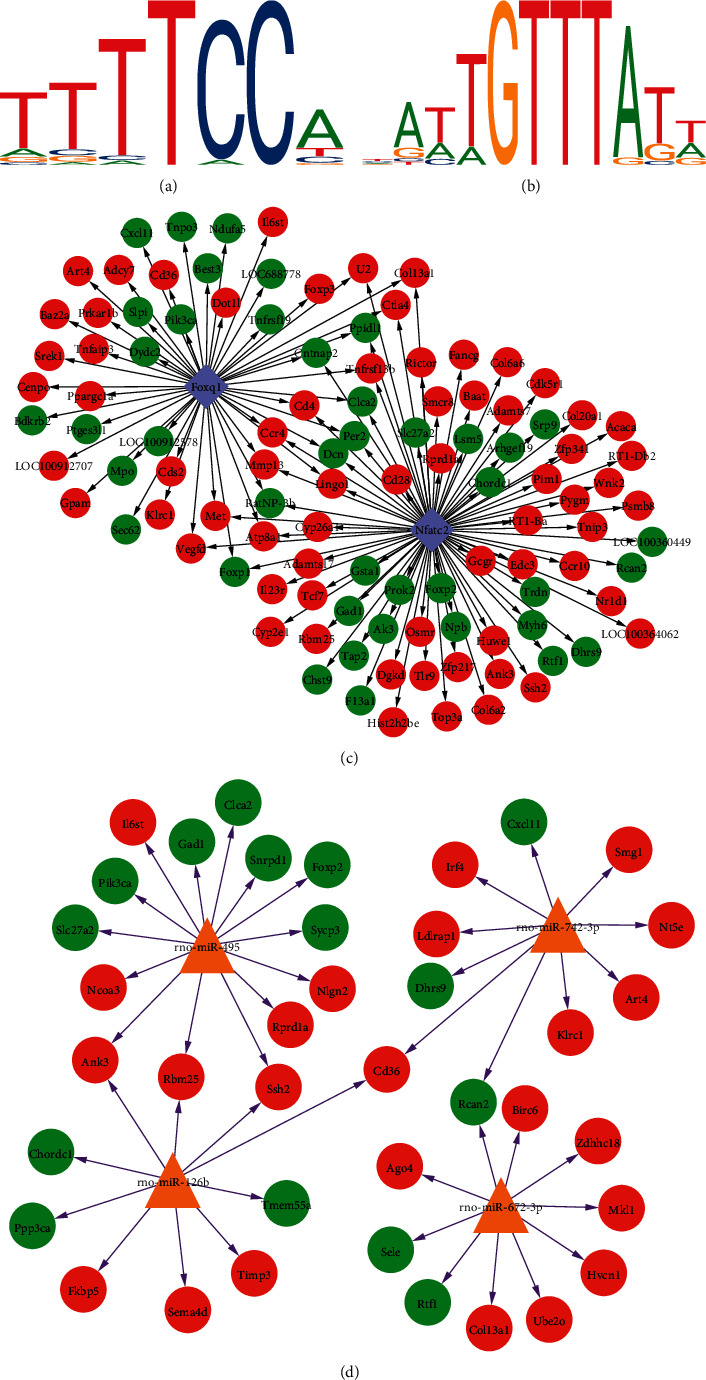
Transcription factor prediction and regulatory network construction. (a) The motif of Nfatc2. (b) The motif of Foxq1. (c) The TF-genes network. The purple diamond represents the upregulated TFs, the red circle represents the upregulated genes, the green circle represents the downregulated genes, and the black arrow represents the regulatory relationship. (d) The miRNA-target regulatory network. The yellow triangle represents miRNA, the red circle represents upregulated genes, the green node represents downregulated genes, and purple arrow represents regulatory relationships.

To explore the regulatory relationship between TF and genes, the TFs of the 874 DEGs were predicted using JASPAR database. Two differentially upregulated TFs, Nfatc2 and Foxq1, were obtained. Their motifs are displayed in Figures 5(a)and 5(b). The transcription factor binding site of Nfatc2 was “TTTTCCA” and of Foxq1 was “*∗∗∗∗*GTTT*∗∗∗*.” In addition, based on the promoter sequence of the protein corresponding genes in PPI network, the binding sites of TFs were identified using the online tool FIMO. A total of 132 binding sites of Nfatc2 were found, which corresponded to 78 genes. Meanwhile, 85 binding sites of Foxq1 were obtained, corresponding to 48 genes. Subsequently, the TF-target regulatory network was established (Figure 5(c)).

**Figure 6:**
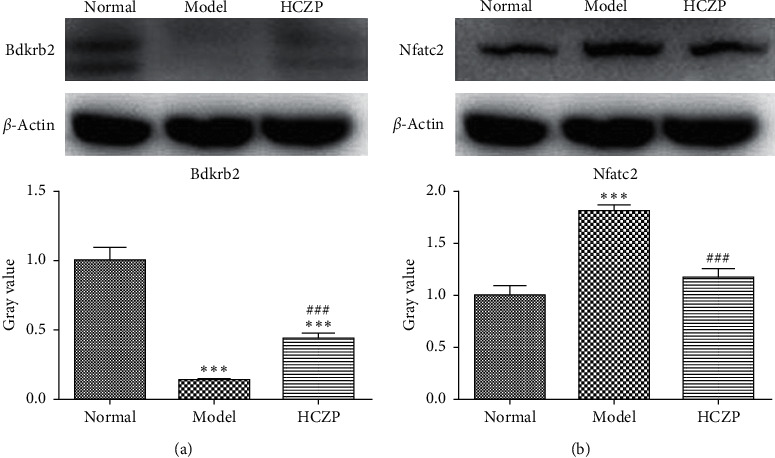
Western blot analysis of Bdkrb2 and Nfatc2 protein expression in rat lung tissues from different groups. (a) Representative western blots showing protein level of Bdkrb2, and protein levels were standardized by *β*-actin. Quantification analysis showed that HCZP increased Bdkrb2 protein level compared with model group. (b) Representative western blot bands of Nfatc2. Quantification analysis showed that Nfatc2 protein level in the HCZP group was lower than in the model group. Data are represented as mean ± SD (*n* = 3). ^*∗∗∗*^*P* < 0.001 compared to normal group. ###*P* < 0.001 compared to the model group.

Using the miRWalk 2.0 database, 1,449 miRNA-target pairs were obtained. Then, miRNAs that simultaneously target at least 10 genes were selected to construct the miRNA-target network. As shown in Figure 6, the miRNA-target gene network was composed of four miRNAs (mo-miR-495, mo-miR-742-3p, mo-miR-126b, and mo-miR-742-3p), 39 target genes, and 44 miRNA-target gene interactions. In this integrated network, five nodes (Ank3, Rbm25, Ssh2, Cd36, and Rcan2) interacted with two miRNAs (Figure 5(d)).

### 3.7. WB and IHC Analysis

In order to verify the accuracy of the transcriptome sequencing results, the protein expression levels of two genes (Bdkrb2 and Nfatc2) were detected using WB and IHC. WB showed that the expression level of Bdkrb2 was significantly lower in the model group than in the normal group (0.139 ± 0.004 vs. 0.999 ± 0.095, *P* < 0.001), while it was markedly higher in HCZP-treated group than in the model group (0.441 ± 0.038 vs. 0.139 ± 0.004, *P* < 0.001) (Figure 6(a)). In addition, the level of Nfatc2 expression was significantly higher in the model group than in the normal group (1.806 ± 0.597 vs. 0.999 ± 0.089, *P* < 0.001), whereas the Nfatc2 level in the HCZP group was observably downregulated compared to that in the model group (1.169 ± 0.081 vs. 1.806 ± 0.597, *P* < 0.001) (Figure 6(b)).

**Figure 7:**
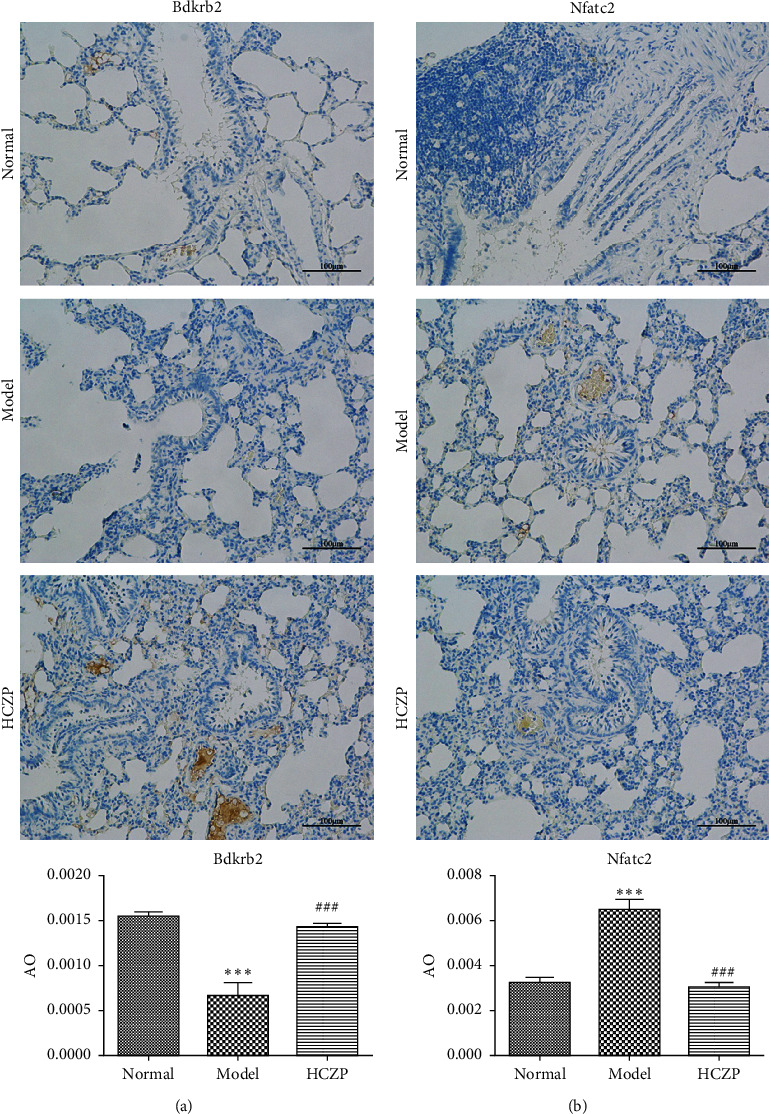
Immunohistochemistry was performed for Bdkrb2 and Nfatc2. (a) Representative IHC staining images of Bdkrb2. Quantitative analysis of the images showed HCZP increased the Bdkrb2 expression compared with model group. (b) Representative IHC staining images of Nfatc2. Quantitative analysis showed HCZP decreased the Nfatc2 expression compared with model group. Data are represented as mean ± SD (*n* = 3). ^*∗∗∗*^*P* < 0.001 versus normal group. ###*P* < 0.001 versus the model group. AO: average optical.

Next, IHC staining was performed to evaluate the expression levels of Bdkrb2 and Nfatc2. The expression level of Bdkrb2 was significantly decreased in the model group, compared with that in the normal group (0.0006 ± 0.0002 vs. 0.00155 ± 0.00008, *P* < 0.001). After HCZP treatment, the expression Bdkrb2 was increased (0.00143 ± 0.00006 vs. 0.0006 ± 0.0002, *P* < 0.001) (Figure 7(a)). Moreover, the expression level of Nfatc2 in the model group was notably higher than that in the normal group (0.0065 ± 0.0007 vs. 0.0033 ± 00004, *P* < 0.001); however, it decreased significantly after HCZP treatment (0.0031 ± 0.00035 vs. 0.0065 ± 0.0007, *P* < 0.001) (Figure 7(b)). These results were consistent with those of RNA sequencing, further confirming the therapeutic effect of HCZP on asthma.

## 4. Discussion

Although asthma can be treated with conventional therapies, such as inhaled corticosteroids and bronchodilators, patients with severe asthma still find the condition intractable. Our study investigated the role of potential genes of HCZP, providing new evidence for molecular therapy and the development of novel drugs. In our analysis, a total of 874 DEGs related to HCZP treatment were screened. Functional enrichment analysis showed that these genes were mainly enriched in GO_BP terms such as immune response and negative regulation of T cell proliferation as well as pathways like CAMs. PPI analysis showed that Cd4 and Bdkrb2 were with higher degrees and might be considered as hub genes. We also screened two TFs (Foxq1 and Nfatc2) and four miRNAs (mo-miR-495, mo-miR-742-3p, mo-miR-126b, and mo-miR-672-3p) with higher degrees in the interaction network. The protein levels of Bdkrb2 and Nfatc2 were verified using WB and IHC, which were in accordance with the results of RNA-Seq.

The CD4 gene encodes the membrane glycoprotein of T lymphocytes and is a receptor for human immunodeficiency virus. This protein functions in initiating or enhancing the early stage of T cell activation and may serve as an important mediator of indirect neuronal injury in immune-mediated disease [40]. In this study, functional enrichment analysis showed that CD4 was notably associated with T cell activation and adaptive immune response. Smith and Larché [41] indicated that T cell activation in the body might lead to manifestations of chronic allergic inflammation, including bronchoconstriction and hyperresponsiveness. In addition, adaptive immune response, a biological process in which antigen-specific T/B lymphocytes activate, proliferate, and differentiate into effector cells after receiving antigen stimulation, is involved in innate and adaptive immunity [42]. Previous study revealed that the expression of CD4 might be increased during inflammation and thrombosis, altering the immune cell-mediated response and leading to atherosclerosis [43]. In addition, Noble et al. [44] found that CD4 were increased in the lamina propria of inflamed inflammatory bowel disease tissue. Furthermore, Lee et al. [45] reported the relationship between primary immunodeficiency (PID) and asthma, indicating that PID was a significant risk factor for asthma exacerbation. These evidences indicated that CD4 was closely involved in immune-mediated disease. However, the relationship between asthma and CD4 gene has not been reported. Taken together, we speculated that CD4 might play a role in asthma by affecting immune-related biological functions.

Another gene, Bdkrb2, was also linked to the asthma treatment. Bdkrb2 encodes the receptor for bradykinins. Among these, 9AA bradykinin causes of a number of reactions, including vasodilation, smooth muscle spasms, and painful fibrous stimulation [46]. Sabatini et al. [47] found that the Bdkrb2 was abnormally expressed in bronchial fibroblasts from asthmatics, indicating that bradykinins were actively involved in the proliferation and differentiation of bronchial fibroblasts, as well as airway remodeling in asthma through MAPK pathway and EGF receptor transactivation. Meanwhile, Ricciardolo et al. [48] revealed that bradykinin is a vasoactive proinflammatory peptide that could activate plasma and tissue kinin, thereby mediating acute inflammatory response of asthma, such as excessive mucus secretion, smooth muscle contraction, and sensory nerve stimulation. In this study, Bdkrb2 was significantly upregulated in the asthma rats after HCZP treatment, suggesting that HCZP might exert therapeutic effects on asthma by affecting the expression of bradykinins.

Several studies reported the role of TFs in the pathogenesis of asthma. In the present study, two TFs, including Foxq1 and Nfatc2, were closely associated with HCZP treatment. Nfatc2, a member of the activated T cell family, is transferred to the nucleus after being stimulated by the T cell receptor and becomes an active T cell transcription complex, which serves a crucial role in inducing gene transcription during the immune response [49]. A previous study demonstrated that the number of T regulatory cells was increased in Nfatc2-deficient mice, inducing immunosuppression to control experimental asthma caused by allergens [50]. In this study, we observed that the protein level of Nfatc2 was significantly decreased after HCZP treatment, suggesting that HCZP may play a therapeutic role by suppressing Nfatc2 expression. In addition, Foxq1 is a member of the Fox gene family known to be involved in cell cycle regulation, cell signaling, and embryonic development [51]. The relationship between Foxq1 and immune-related diseases has been explored. Ovsiy et al. [52] indicated that Foxq1 participated in the development of chronic inflammatory disease atopic dermatitis by stimulating monocyte movement and increasing proinflammatory potential. However, no studies have reported the roles of Foxq1 in asthma; further studies with biological experiments are needed to illustrate its exact molecular mechanism.

In addition to TFs, miRNAs also serve important roles in the pathogenesis of asthma [53, 54]. In this study, we identified four miRNAs (mo-miR-495, mo-miR-742-3p, mo-miR-126b, and mo-miR-672-3p) that were involved in asthma treatment. Studies indicated that miR-495 participated in the regulation of inflammatory response, which acted as a negative modulator of inflammation-motivating pathways [55, 56]. miR-742-3p was only reported to be involved in excessive lipid deposition [57], and miR-126b was essential for lymphatic system development in mammals [58]. In addition, no article reported the relationship between miR-672-3p and diseases. Unfortunately, these miRNAs have not been previously documented in asthma; therefore, the roles of these identified genes in asthma need to be investigated in further research.

Modern genomics and proteomics tools have revealed potential therapeutic antisense targets for asthma, providing a theoretical basis for the development of anti-mRNA drugs [59]. A previous study indicated that RNA-based gene silencing strategy could not only be used as a research tool, but also as a potential therapeutic intervention for allergic asthma [60]. In this study, we identified several genes that were associated with the development of asthma, and these are potential molecular therapeutic targets for asthma. However, our study had two main limitations. First, not all of the identified potential hub genes have been verified. Second, we found that miRNAs were related to asthma treatment, but did not clarify their mechanism of action. Therefore, these candidate biomarkers need to be further validated using a larger sample population before they can be applied in clinical therapy.

## 5. Conclusion

Based on transcriptome analysis, this study demonstrated that HCZP alleviated the symptoms of asthma through various mechanisms. Results revealed that the immune response and T cell differentiation-related genes contributed to the therapeutic effect of HCZP against asthma. Our findings could help identify novel and effective therapeutic targets and provide evidence for a better understanding of asthma pathology.

## Figures and Tables

**Table 1:** Top 5 GO terms and KEGG pathways of DEGs.

Category	Term	Count	Genes	*P* value
GO_BP	GO:0006955∼immune response	28	LOC100910650, CSF2, RT1-DB2, CXCL3, CXCL11, RT1-BA, RT1-BB, TLR9, LOC100361009, CCR10, ZAP70, RT1-CE10, CD28, RT1-CE3, RT1-CE6, RGD1563231, RT1-CE5, CTLA4, LOC100912707, RT1-A2, RT1-A1, TNFRSF10 B, CD36, CCR4, CD274, SLPI, TNFAIP3, BMP6	3.30*E* − 07

GO_BP	GO:0042116∼macrophage activation	5	CRTC3, CSF2, SLC7A2, AIF1, FOXP1	2.21*E* − 04

GO_BP	GO:0042130∼negative regulation of T cell proliferation	8	PDE5A, CD274, PLA2G2A, CTLA4, RT1-BA, FOXP3, SHH, RT1-BB	2.72*E* − 04

GO_BP	GO:0002474∼antigen processing and presentation of peptide antigen via MHC class I	7	RT1-A2, RT1-CE3, RT1-A1, RT1-CE6, RT1-CE5, RT1-CE10, RT1-T24-3	0.00112

GO_BP	GO:0000122∼negative regulation of transcription from RNA polymerase II promoter	42	TSHZ1, BACH2, TFCP2L1, FST, MITF, CBX4, MYEF2, AURKB, ANKRD1, CBFA2T3, SHH, EDNRB, NR1D1, RTF1, PER2, ZFP217, NFATC2, ETV3, MDFI, TCF7, BEND3, LOC100912068, IKZF1, MET, ASXL1, SKI, ZFP148, FOXP3, LPIN1, FOXP1, FOXP2, SUZ12, HOXB3, HOXB4, DACT1, CD36, PSEN1, HES5, HIPK2, SEMA4D, HDAC7, BMP6	0.00128

GO_CC	GO:0005615∼extracellular space	69	NRG4, PXDN, IL6ST, AMN, CXCL11, TPBGL, SHH, LOC100361009, RATNP-3B, OGN, SERPINE2, SERPINE3, SOSTDC1, RGD1305645, EPPIN, CPA3, SPON1, HYAL1, STC2, ACTA2, MMP13, LOC10091270,LYZL1, VEGFD, CD36, KLHL17, HIST2H2BE, SERPINB7, PLA2G2A, SLPI, COL1A1, SEMA4D, SCGB3A1, RGD1359108, CSF2, BPIFB1, ADAMTS13, CXCL3, SSH2, JCHAIN, CCL9, DCN, TIMP3, ABI3BP, FBRS, CPZ, COL6A2, ANGPTL1, SCG2, TMC8, IL1RL1, RGD1563231, MET, SELENOP, CHI3L1, NLGN2, SERPINI1, CCL11, LCN2, SBPL, S100 B, C1RL, MPO, IGFBP2, TPSAB1, AGR2, SELE, COL20A1, BMP6	1.73*E* − 04

GO_CC	GO:0009897∼external side of plasma membrane	22	OSMR, IL1RL1, VTCN1, SELL, IL6ST, TNFRSF13 B, CTLA4, RT1-BA, IL7R, SPA17, LOC100361105, RT1-BB, RT1-A2, CD36, CCR4, CD274, CHRNA4, CHRNA7, CD4, KLRC1, CD28, CD200R1	2.38*E* − 04

GO_CC	GO:0042612∼MHC class I protein complex	6	RT1-A2, RT1-CE3, RT1-A1, RT1-CE6, RT1-CE5, RT1-CE10	8.07*E* − 04

GO_CC	GO:0005578∼proteinaceous extracellular matrix	20	COL4A3, ADAMTS17, IL1RL1, ADAMTS13, SPOCK2, DCN, TPBGL, TIMP3, MMP13, ABI3BP, COL5A1, SHH, CPZ, ADAMTS7, OGN, COL6A6, COL6A2, TNN, COL1A1, SPON1	8.13*E* − 04

GO_CC	GO:0043198∼dendritic shaft	8	HCN2, PSEN1, PREX1, SLC12A5, NLGN2, CHRNA7, GPER1, SYNGAP1	0.00358

GO_MF	GO:0042605∼peptide antigen-binding	8	RT1-CE3, RT1-A1, TAP2, RT1-CE10, RT1-BA, SLC7A5, RT1-BB, RT1-T24-3	2.01*E* − 04

GO_MF	GO:0004872∼receptor activity	14	EXTL3, IZUMO1R, TNFRSF13 B, CTLA4, TNFRSF17, NLGN2, FPR3, PAQR4, EDNRB, ITGB7, TNFRSF19, ANTXR1, SEMA4D, CD200R1	2.77*E* − 04

GO_MF	GO:0004924∼oncostatin-M receptor activity	3	OSMR, IL6ST, LIFR	0.00331

GO_MF	GO:0042800∼histone methyltransferase activity (H3-K4 specific)	5	KMT2A, SETD1B, KMT2C, DYDC1, DYDC2	0.00399

GO_MF	GO:0005539∼glycosaminoglycan binding	5	SERPINE2, SPOCK2, DCN, ABI3BP, SHH	0.00480

KEGG_PATHWAY	rno04514: cell adhesion molecules (CAMs)	23	CLDN8, CLDN7, RT1-CE3, VTCN1, SELL, RT1-CE5, CTLA4, NLGN2, NTNG2, RT1-BA, CDH4, RT1-BB, RT1-A2, RT1-A1, GLYCAM1, ITGB7, CD274, RT1-CE10, CD4, SELE, SELPLG, CD28, RT1-T24-3	1.51*E* − 08

KEGG_PATHWAY	rno04512: ECM-receptor interaction	12	COL4A3, COL4A2, GP6, CD36, COL6A6, ITGB7, COL6A2, ITGB5, TNN, COL1A1, COL5A3, COL5A1	9.32*E* − 05

KEGG_PATHWAY	rno04612: antigen processing and presentation	12	LOC103692716, RT1-A2, RT1-CE3, RT1-A1, TAP2, RT1-CE5, RT1-CE10, CD4, RT1-BA, KLRC1, RT1-BB, RT1-T24-3	2.04*E* − 04

KEGG_PATHWAY	rno04940: type I diabetes mellitus	10	RT1-A2, RT1-CE3, RT1-A1, RT1-CE5, RT1-CE10, RT1-BA, GAD1, RT1-BB, RT1-T24-3, CD28	5.59*E* − 04

KEGG_PATHWAY	rno05320: autoimmune thyroid disease	10	RT1-A2, RT1-CE3, RT1-A1, RT1-CE5, RT1-CE10, CTLA4, RT1-BA, RT1-BB, RT1-T24-3, CD28	6.16*E* − 04

**Table 2:** The topological property score of top 10 nodes in PPI network.

Gene name	Degree	Gene name	Betweenness	Gene name	Closeness
Hvcn1	13	Csf2	9536.876	Csf2	0.00722
Bdkrb2	12	LOC680121	8310.523	Il7r	0.00722
Ccl9	12	Pik3ca	7250.114	Cd4	0.00722
LOC680121	12	Il7r	6126.990	LOC680121	0.00721
Col1a1	12	Cd4	5988.467	Cd274	0.00721
Fabp5	11	Met	5808.000	Ldlrap1	0.00721
Hist2h2be	11	Dcn	5656.000	Snap91	0.00721
Foxp3	11	Col1a1	5520.000	Foxp3	0.00721
Cxcl3	10	Gad1	5443.524	RatNP-3b	0.00720
Cd4	10	RatNP-3b	5180.190	Pik3ca	0.00720

## Data Availability

The raw data supporting the conclusions of this manuscript will be made available by the corresponding author, without undue reservation, to any qualified researcher.
